# 2300: E-prescribing research participation: Feasibility of recruiting research participants using an EMR-integrated health information technology

**DOI:** 10.1017/cts.2017.36

**Published:** 2018-05-10

**Authors:** Gillian Feldmeth, Leidy Gutierrez, Stacy Tessler Lindau, Jennifer A. Makelarski, Edward T. Naureckas, Julian Solway

## Abstract

OBJECTIVES/SPECIFIC AIMS: To study the rate of recruitment to the Pulmonary Research Registry (PRR) at the University of Chicago using HealtheRx recruitment Versus usual practice. METHODS/STUDY POPULATION: CommunityRx is a health information technology, integrated with electronic medical record (EMR) platforms, that generates personalized referrals (“HealtheRxs”) for community-based programs and services that address basic and other health-related self-care needs. The target population included people ages 18 and older, English speaking, living in 1 of 16 ZIP codes on Chicago’s south and west sides (106 mi^2^) who received care at ≥1 of 28 CommunityRx partner sites and had a diagnosis of asthma or COPD. Between December 2015 and December 2016, information about pulmonary research participation opportunities was included on the HealtheRxs of eligible patients contemporaneously with usual registry recruitment methods. Usual methods, used since 2010 by the PRR group, included public advertisements requiring the patient to call the research team for more information and education of eligible patients identified during routine clinical care with a Pulmonary/Critical Care clinician or when enrolling in a pulmonary clinical trial. We hypothesized that, compared with usual recruitment practices, the HealtheRx recruitment strategy would increase the rate and decrease the per subject cost of patient recruitment to a prospective registry. Total annual recruitment costs for each method were calculated and divided by the number of consented PRR enrollees per method. RESULTS/ANTICIPATED RESULTS: Between December 22, 2015 and December 15, 2016 13,437 HealtheRxs (8762 for asthma, 3842 for COPD, and 833 for both asthma and COPD) were generated with the recruitment advertisement. In total, 41 patients responded to the ad and participated in the phone survey. In which 15 (36.5%) participants self-reported a diagnosis of asthma only (65% of all HealtheRxs with advertisement were for asthma only), 9 (22%) reported a diagnosis of COPD only (28.5% of all HealtheRxs with advertisement were for COPD only), and 17 (41.5%) reported diagnoses of both asthma and COPD (6.2% of all HealtheRxs with advertisement were for asthma and COPD). Most participants were female (n=28), non-Hispanic black (n=37), and not employed (n=39). The median age was 57. The majority (n=31) had never participated in health or medical research and was not aware of current opportunities to participate in research (n=25). All 41 participants expressed interest in joining PRR and were mailed a blank PRR consent form and a prepaid return envelope with their incentive check for the telephone survey. To date, 5 participants returned a signed consent form via mail and were enrolled in PRR. During the same period, 4 patients enrolled in PRR via usual recruitment methods. The cost per subject to enroll in PRR was $364.40 using the HealtheRx recruitment and $4590 using usual practice. DISCUSSION/SIGNIFICANCE OF IMPACT: NIH has called for innovation in research recruitment methodologies to increase enrollment especially of people who are under-represented in clinical trials research. This study demonstrates the feasibility and efficiency of using an EMR-integrated recruitment method to enroll people of under-represented minority groups to a research registry.

